# Beyond Getting Rid of Stupid Stuff in the Electronic Health Record (Beyond-GROSS): Protocol for a User-Centered, Mixed-Method Intervention to Improve the Electronic Health Record System

**DOI:** 10.2196/25148

**Published:** 2021-03-16

**Authors:** Ahmed Umar Otokiti, Catherine K Craven, Avniel Shetreat-Klein, Stacey Cohen, Bruce Darrow

**Affiliations:** 1 Department of Medicine Icahn School of Medicine at Mount Sinai Mount Sinai Health System New York, NY United States; 2 Clinical Informatics Group, Information Technology Department Icahn School of Medicine at Mount Sinai Mount Sinai Health System New York, NY United States; 3 Institute for Health Care Delivery Science, Department of Population Health Science and Policy Icahn School of Medicine at Mount Sinai Mount Sinai Health System New York, NY United States; 4 Department of Rehabilitation and Human Performance Icahn School of Medicine at Mount Sinai Mount Sinai Health System New York, NY United States; 5 Department of Cardiology and Health Information Technology Mount Sinai Health System New York, NY United States

**Keywords:** electronic health records, burnout, psychological, user-centered design, usability, EHR optimization

## Abstract

**Background:**

Up to 60% of health care providers experience one or more symptoms of burnout. Perceived clinician burden resulting in burnout arises from factors such as electronic health record (EHR) usability or lack thereof, perceived loss of autonomy, and documentation burden leading to less clinical time with patients. Burnout can have detrimental effects on health care quality and contributes to increased medical errors, decreased patient satisfaction, substance use, workforce attrition, and suicide.

**Objective:**

This project aims to improve the user-centered design of the EHR by obtaining direct input from clinicians about deficiencies. Fixing identified deficiencies via user-centered design has the potential to improve usability, thereby increasing satisfaction by reducing EHR-induced burnout.

**Methods:**

Quantitative and qualitative data will be obtained from clinician EHR users. The input will be received through a form built in a REDCap database via a link embedded in the home page of the EHR. The REDCap data will be analyzed in 2 main dimensions, based on nature of the input, what section of the EHR is affected, and what is required to fix the issue(s). Identified issues will be escalated to relevant stakeholders responsible for rectifying the problems identified. Data analysis, project evaluation, and lessons learned from the evaluation will be incorporated in a Plan-Do-Study-Act (PDSA) manner every 4-6 weeks.

**Results:**

The pilot phase of the study began in October 2020 in the Gastroenterology Division at Mount Sinai Hospital, New York City, NY, which includes 39 physicians and 15 nurses. The pilot is expected to run over a 4-6–month period. The results of the REDCap data analysis will be reported within 1 month of completing the pilot phase. We will analyze the nature of requests received and the impact of rectified issues on the clinician EHR user. We expect that the results will reveal which sections of the EHR have the highest deficiencies while also highlighting issues about workflow difficulties. Perceived impact of the project on provider engagement, patient safety, and workflow efficiency will also be captured by evaluation survey and other qualitative methods where possible.

**Conclusions:**

The project aims to improve user-centered design of the EHR by soliciting direct input from clinician EHR users. The ultimate goal is to improve efficiency, reduce EHR inefficiencies with the possibility of improving staff engagement, and lessen EHR-induced clinician burnout. Our project implementation includes using informatics expertise to achieve the desired state of a learning health system as recommended by the National Academy of Medicine as we facilitate feedback loops and rapid cycles of improvement.

**International Registered Report Identifier (IRRID):**

PRR1-10.2196/25148

## Introduction

### Background

Substantial evidence indicates that electronic health records (EHRs) contribute greatly to clinician burnout [1-5]. This burden arises from factors like EHR usability or lack thereof, perceived loss of autonomy, and documentation; this leads to less clinical time with patients and clinicians creating various workarounds to the problem with the associated potential to compromise execution of care consistent with patient safety and quality [1,6]. Clinician burnout and dissatisfaction can adversely impact provider engagement, which in turn, can negatively impact patient safety and health care quality [7,8].

In an effort to alleviate the EHR burden imposed on clinicians and its adverse consequences on the quality of health care delivery, Hawaii Pacific Health implemented a program called Getting Rid of Stupid Stuff (GROSS). In this program, clinicians were asked to come forward with anything in the EHR that they thought was poorly designed, unnecessary, or just “plain stupid” [9].

This protocol is a description of how we plan to implement a modified and extended version of the Hawaii Pacific Health system's GROSS program in our medical center at Mount Sinai Hospital in New York City, NY. Our program will be called Beyond Getting Rid of Stupid Stuff (Beyond-GROSS).

### Hawaii Pacific Health GROSS Experience

Hawaii Pacific Health is the largest private health care organization in Hawaii with 4 medical centers, 592 beds, 6950 staff, and 1764 physicians [10]. The GROSS program seeks direct input from clinicians about various aspects of the EHR and documentation that is not useful or counterintuitive.

Categories of requests received included the following: documentation that was never meant to occur and would require little consideration to eliminate or fix, documentation that was needed but could be completed in a more efficient or effective way with newer tools or better understanding, and documentation that was required but for which clinicians did not understand the requirement or tools available to them.

After 1 year of implementation, a total of 188 requests were received in all 3 categories by the GROSS team. Suggestions from other disciplines were not presented. Also, suggestions related to issues other than EHR improvement were not permitted [9].

The results showed the following: There were more responses from nurses (n=146) than physicians (n=42), the vendor (Epic EHR) was supportive of the effort, there was an overall organizational acceptance of the project by clinicians, and there were minor changes in the EHR based on suggestions from users. Data about physician engagement were pending at the time of publication.

### Mount Sinai Beyond-GROSS Program Objectives and Justification

The issue of burnout and clinician engagement is now a widespread concern among all stakeholders, including government and expert organizations, and the concept of the Quadruple Aim of health care delivery has been widely adopted across institutions [11-13]. The Quadruple Aim was born out of the need to add provider satisfaction and well-being to the initial Institute of Health Improvement's Triple Aim of better health, lower cost, and better care [12].

**Figure 1:**
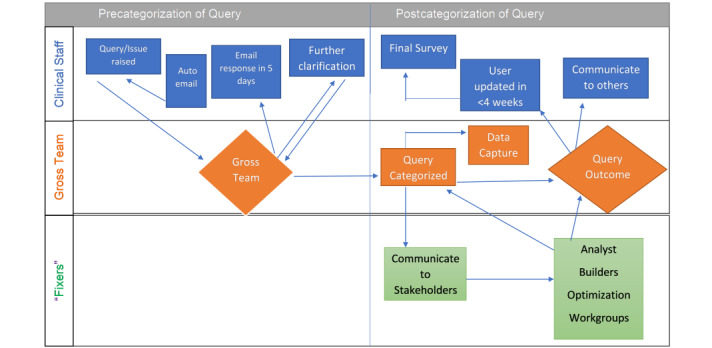
Beyond-Getting Rid of Stupid Stuff (GROSS) flow chart.

Apart from the contributions of technology (EHR) to the dissatisfaction of clinicians, the sociocultural and work processes also play an important role in the determination of successful implementation. The interplay of technology and process efficiency described in sociotechnical constructs is very important for overall success [14-18]. In essence, a superb technology application or EHR deployed in an inefficient system will not succeed [19]. Based on this premise, our Beyond-GROSS project will involve soliciting feedback from clinicians about workflow and process issues in addition to EHR-specific problems (see Figure 1 for a diagram of overall project workflow).

Beyond-GROSS dovetails with our existing EHR governance structures and other efforts of the Mount Sinai Health System to alleviate the issue of EHR burden through its various EHR optimization workgroups. Apart from the required initial mandatory online EHR training for all new clinicians at Mount Sinai Health System, there are governance mechanisms to increase EHR use proficiency among clinicians and to optimize and standardize the EHR configuration, navigation, and content. These include ambulatory and inpatient EHR optimization working groups, which are collaboratively led by members of the Clinical Informatics Group and Information Technology (IT). The Clinical Informatics Group is a unit reporting to the Chief Medical Information Officer (CMIO). These working groups are comprised of members from many clinical departments and lead analysts from the EHR applications teams. Clinicians can bring EHR change requests to these bodies through their department’s members. The working groups discuss proposed changes, seek additional departmental feedback as needed, and make decisions. Then, the analyst representatives ensure that build changes are made. Mount Sinai Health System also operates an EHR physician champion program whereby the physician champions in each department serve as resource persons for other clinicians who need help using and navigating the EHR. In many cases, these departmental physician champions are also representatives to the EHR working groups.

Our intervention adds the first mechanism through which clinicians can directly report issues via the EHR. It expands request categories and captures more granular aspects of requests. The intervention expedites fast fixes where possible and appropriate channeling for resolution. The Beyond-GROSS team members, a multidisciplinary subset of the Clinical Informatics Group including the Deputy CMIO, triage reported issues for type of resolution needed (eg, user education, EHR quick fix, potential workflow issue with EHR component); appropriately channel requests outside of the team as needed, including directly to Epic analysts and existing EHR working groups; and guide requests through to resolution.

## Methods

### Intervention Setting and Population

The program will be implemented in the Mount Sinai Health System, New York City, NY, which is composed of 8 hospital campuses, 13 ambulatory surgical centers, and over 6600 primary and specialty care physicians. We plan to start by conducting a pilot program in a service line of the main campus of the Mount Sinai Hospital and gradually scale the project across the health system over 2 years.

Participants in the pilot will be comprised of clinicians in our academic gastroenterology practice spanning inpatient, outpatient, and endoscopy services, which includes 25 attendings, 14 fellows, and 5 nurses, with a patient volume of more than 6000 endoscopy procedures and 26,800 outpatient visits. This division is an excellent pilot group because it has an engaged workforce comfortable with using technology and a focus on wellness and quality, and it is comprised of a variety of team members across 3 key settings who are all using the EHR to support care delivery.

### Intervention Design and Procedure

The overall design will be mixed methods utilizing both qualitative and quantitative methods of data gathering and analysis in a Plan-Do-Study-Act (PDSA) iterative approach of quality improvement.

The project will solicit suggestions and input from front-end clinicians (physicians and nurses) about the EHR and workflow issues they encounter during their daily tasks. They will be also encouraged to come forth with suggested ways of overcoming the issues raised. Our initial pilot will take place over a duration of 4-6 months with the intention to implement the program throughout our health system over a period of 2 years. Lessons learned from data analysis and project evaluation will be incorporated in a PDSA fashion every 8 weeks as we scale throughout the organization.

### Data Collection Tool, Data Management, and Analysis

**Figure 2:**
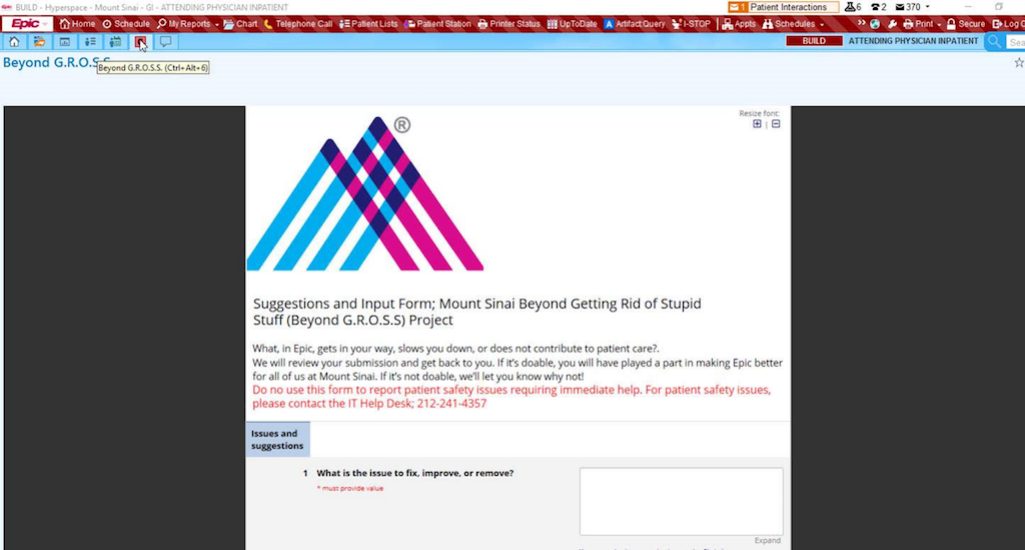
Beyond-Getting Rid of Study Stuff (GROSS) link embedded in the electronic health record (EHR).

We will be soliciting input from clinicians through a form built in the REDCap data capture application with a link to the form conveniently located in the homepage of the EHR for easy access when they encounter documentation that can be optimized (see Figure 2 for Beyond-Gross icon “G” embedded in the EHR) [20]. Clicking the icon opens the form in a browser for data input by the clinician. The G icon was embedded in the EHR (Epic EHR) with the help of an in-house Epic analyst (see Figure 2 for the “G” icon in EHR with link to the REDCap database).

The technicalities involve creating a new activity in text located at the top tab. Parameters like link title and URL were set, and filters were placed limiting the activity to only the Gastroenterology Division. The new activity was also added to the desired roles so that only physicians and nurses in the designated department would see the Beyond-GROSS link “G” in their top tab once they log into Epic.

The data from the form are then automatically stored in our health system’s secure instance of the REDCap database for further analysis by our team. The REDCap form is made up of 2 sections: The first section contains questions relating to the potential issues raised by the frontline clinician and specific issue, location where the issue occurred, and best way to follow up with requesters (see Textbox 1 for form content and description); this corresponds to questions 1 through 11 in the form. The second aspect of the form is for completion by an assigned Beyond-GROSS team member for further analysis of the requester’s input. This section was set up in REDCap to be visible only to an authorized Beyond-GROSS team member for the purpose of analysis; this corresponds to questions 12 through 33 of the survey (Table 1). A sample of the overall request instrument can be found in Multimedia Appendices 1-4.

Follow-up on input received and analysis of data input include the items listed in Textbox 2.

**The contents of the REDCap form section that will be filled out by a clinician requester.**

1. Issues and suggestions:
  What is the issue to fix, improve, or remove?
  Please upload any supporting document or screenshot.
  Why is it beneficial to fix or improve the issue?
  Please share any suggestions on how to fix or improve this issue.
2. Contact and location of clinician requester:
  What is your name?
  What is your email address?
  What is the best phone number to contact you?
  Where do you work? (hospital/department/clinic)
  What EPIC login department did you use when you encountered this issue?
  What is your clinical role? (registered nurse [RN], physician, physician assistant [PA], nurse practitioner [NP])

**Table 1:** The contents of the REDCap form section that will be filled out by a designated Beyond-Getting Rid of Study Stuff (GROSS) team member.

Sections	Specific form questions
Team member	Name of team member handling the issues raised by requester
EHR^a^ section concerned	Flowsheet, Smart tools, Orders, BPA^b^, Profiles, etc.
Categorization of issues: nature of the issue	Problem inadvertently created, tools not available, requester unaware of tools, etc.
Categorization of issues: what is required to fix it	Easy fix no build required, requires approval, requester use-optimization needed, not feasible, etc.
Updates	Status of request resolution and communication with users until final resolution and closure

^a^EHR: electronic health record.

^b^BPA: best practice advisory.

**Process to follow up on requests and communicate with users.**

1. An automated email response to the submission received in REDCap will be sent to the requester acknowledging the receipt of the issue noted.
2. Beyond-Getting Rid of Stupid Stuff (GROSS) team members will then proceed with analysis of the request with the intention to categorize the request input for action based on a predetermined categorization scheme (Textbox 1).
3. A personalized email response from one of the Beyond-GROSS team members acknowledging receipt of the REDCap submission and to provide updates on possible solutions to the issue will be sent to the requester within 5 business days.
4. Further clarification may be required from clinician requesters about issues raised in the request via:
  - Email exchanges between the project team members and requester
  - Other basic qualitative methods may be needed for further understanding depending on request type:
    Personal interview with requester
    Walk through of affected unit to observe workflow
    Field study or observation of affected unit
    Diary studies
    Focus group
5. Request classification by Beyond-GROSS team members for action and issues or requests will be categorized based on:
  - The nature of the request
  - Requester unaware of extant tools/resources to undertake task efficiently
  - Requester aware of extant tools but not proficient at using tools/resources
    Tools/resources for task not available
  - Tools/resources exist but improvements are needed to undertake clinician's task efficiently
  - Never meant to occur/inadvertent
    Workflow/process optimization required; no direct electronic health record (EHR) fix
  - What is required to address/fix issues
    Easy fix; minimal/no build required; no patient safety consequences if stopped
  - Approval by the hospital’s regulatory and patient safety committee needed
    Newer tools or build needed
  - User optimization or training needed
  - Not feasible to fix
  - Workflow or process reengineering is needed
    Requires multiple dimensions to fix; both workflow re-engineering plus any other EHR issues (see above)
6. After team categorization of a request, issues will be escalated to the respective stakeholders concerned for resolution of the issue. Such stakeholders include EHR analysts, the safety and regulatory committee, unit leadership, information technology leadership, etc.
7. A follow-up email will be sent to the requester by a designated Beyond-GROSS team member to give an update on the request input within 4 weeks of filling out the request form:
  Request not feasible and why
  Feasible and work in process with estimated time to complete
  Feasible but unable to proceed for now and reason stated
  Completed and resolved

In the event the issue is not resolved within 4 weeks of filling out the request form, the requester will be given a monthly update of the ongoing work on the issue raised until final resolution is reached.

### Data Capture After Query Categorization and Resolution

Apart from assisting with internal audit and quality improvement purposes, we hope to share lessons learned and quality improvement achieved in standard publications and presentations. We will be analyzing both quantitative and possible qualitative data that results from the intervention.

#### Qualitative Data

Qualitative data will be collected using multiple methods. A postresolution survey will be conducted with requesters to solicit suggestions to improve the project and information on the impact of the project on provider and staff engagement. Iterative transcriptions and data will be obtained from requester interviews or focus group inquiry conducted as part of our PDSA cycles depending on the nature of participants’ requests. Iterative transcriptions from any further qualitative activities carried out to understand issues raised (eg, interviews, field observations) will also be recorded. In the event an interview is conducted, the interviews will be transcribed verbatim. The data found in the interviews will be categorized and analyzed using manual thematic analysis [21]. We will also collect data on clinician satisfaction and the impact on engagement.

#### Quantitative Data

Quantitative data will also be collected using multiple methods, including direct data from our project database and the amount and percentage of requests in each query category, based on the nature of the query and what is required to address issues: percentage of requests directly related to the EHR and percentage of workflow issues not directly related to the EHR.

As applicable and when possible, we will attempt to capture the impact of solution(s) resulting from the Beyond-GROSS project after a predetermined duration of implementation: impact on cost reduction, reduction in errors, reduction in time on the task to figure out baseline before the issue was fixed and repeated after implementation of fixes to determine impact, increase in patient satisfaction where applicable, and other indices as dictated by the specific issue fixed.

Quantitative data will be analyzed using SPSS 20.0 (IBM Corp, Armonk, NY).

### Ethics and Governance

The project will be conducted according to the ethical guidelines of the Helsinki declaration [22]. The study is a quality improvement project. The Icahn School of Medicine Institutional Review Board (IRB # 20-04212) determined that it did not need institutional review board review.

## Results

We have formed our team, the project charter has been inaugurated, and the CMIO and other IT leaders have approved the project. The leadership approval also grants the Beyond-GROSS team access to utilize our IT service help desk, which we use to engage members of Epic analyst teams and other IT teams as needed to resolve EHR issues. The pilot will run for 4-6 months with formative evaluation and potential adjustments every 4 weeks. Logistic discussions with the Gastroenterology Division have been completed. Our EHR analysts have embedded the REDCap form link in the home page of the HER, and the project launched in October 2020. Lessons learned from the pilot phase will help us scale to the whole enterprise in phases based on available resources.

The data obtained from the project will provide us with information about aspects of our EHR that hinder efficiency. This information will be useful to our EHR vendor as they plan subsequent upgrades to make improvements. Data will also show us areas in the EHR for which requesters are not using the available tools to efficiently undertake their work. This information will be highly useful to our EHR optimization team because it will assist in determining areas of priority during future training and optimization sessions. Data on time-to-response from the various committees and stakeholders will serve as a means of an internal audit of our informatics governance structure. The direct impact of project on quality will also be sought.

The outcome of most of the issues fixed may not be immediately quantifiable. Also, we are unable to know ab-initio which issues requesters will report, and as such, we are unable to make a specific determination about indices to quantify improvement. However, we hope to incorporate lessons learned from the pilot project to arrive at possible indices for quantifying quality improvement.

We intend to disseminate this information to stakeholders through research or quality improvement forums and peer-reviewed journals.

## Discussion

Many clinicians find it undesirable and time consuming to call the IT helpdesk for IT-related or workflow issues encountered during clinical care, mostly due to long wait times when the health system’s IT helpdesk is contacted for EHR-specific inquiries [23]. This project will assist in mitigating identified factors that encourage workaround behaviors amongst clinicians, which include perceived or real workflow blocks, and problematic rules that have outlived usefulness, hindering smooth operation; poor workflow design; and other issues [6].

Small tweaks may improve efficiency and reduce burnout in the long run while also improving patient safety. This project may give rise to workflow reengineering, since sociotechnical and process issues also play an important role [19]. The process of getting direct input from staff and clinicians in this project may increase employee engagement, morale, and satisfaction. Most clinicians are delighted to be part of positive force of change, and implementing employee feedback increases sense of belonging and job satisfaction [7,24].

We hope to reap all the benefits of the highest level of health care usability and human-centeredness maturity model as proposed by the Healthcare Information Management System Society and increase individual and organizational efficiency [25].

We will also be using informatics to achieve the desired state of a learning health system as recommended by the National Academy of Medicine as we facilitate feedback loops and rapid cycles of improvement [26].

Although an earlier version of the Beyond-GROSS project achieved high clinician satisfaction in the implementation at Hawaii Pacific, it is not without its limitations. The inability to identify objective quantitative indices prior to implementation is an identified limitation. Another limitation is that it may be challenging to implement in a center with resource limitations: personnel, database, and IT infrastructure.

## Appendix

Multimedia Appendix 1 BEYOND-GROSS survey instrument Page 1.

Multimedia Appendix 2 Beyond-GROSS survey instrument page 2.

Multimedia Appendix 3 Beyond-GROSS survey instrument page 3.

Multimedia Appendix 4 Beyond-GROSS survey instrument page 4.

## Abbreviations

CMIO: Chief Medical Information Officer
EHR: electronic health record
GROSS: Getting Rid of Stupid Stuff
IT: information technology
PDSA: Plan-Do-Study-Act

## References

[1] Khairat S, Burke G, Archambault H, Schwartz T, Larson J, Ratwani R (2018). Perceived Burden of EHRs on Physicians at Different Stages of Their Career. Appl Clin Inform.

[2] Patel RS, Bachu R, Adikey A, Malik M, Shah M (2018). Factors Related to Physician Burnout and Its Consequences: A Review. Behav Sci (Basel).

[3] Reith TP (2018). Burnout in United States Healthcare Professionals: A Narrative Review. Cureus.

[4] Melnick ER, Dyrbye LN, Sinsky CA, Trockel M, West CP, Nedelec L, Tutty MA, Shanafelt T (2020). The Association Between Perceived Electronic Health Record Usability and Professional Burnout Among US Physicians. Mayo Clin Proc.

[5] Robertson SL, Robinson MD, Reid A (2017). Electronic Health Record Effects on Work-Life Balance and Burnout Within the I Population Collaborative. J Grad Med Educ.

[6] Debono DS, Greenfield D, Travaglia JF, Long JC, Black D, Johnson J, Braithwaite J (2013). Nurses' workarounds in acute healthcare settings: a scoping review. BMC Health Serv Res.

[7] Brand SL, Thompson Coon J, Fleming LE, Carroll L, Bethel A, Wyatt K (2017). Whole-system approaches to improving the health and wellbeing of healthcare workers: A systematic review. PLoS One.

[8] Fred HL, Scheid MS (2018). Physician Burnout: Causes, Consequences, and (?) Cures. Tex Heart Inst J.

[9] Ashton M (2018). Getting Rid of Stupid Stuff. N Engl J Med.

[10] Herman B (2014). Outpatient volume fuels Hawaii Pacific Health's FY14 gains. Modern Healthcare.

[11] (2020). Strategy on Reducing Burden Relating to the Use of Health IT and EHRs. The Office of the National Coordinator for Health Information Technology (ONC).

[12] Bodenheimer T, Sinsky C (2014). From triple to quadruple aim: care of the patient requires care of the provider. Ann Fam Med.

[13] Sikka R, Morath JM, Leape L (2015). The Quadruple Aim: care, health, cost and meaning in work. BMJ Qual Saf.

[14] Li J (2010). A Sociotechnical Approach to Evaluating the Impact of ICT on Clinical Care Environments. Open Med Inform J.

[15] Black AD, Car J, Pagliari C, Anandan C, Cresswell K, Bokun T, McKinstry B, Procter R, Majeed A, Sheikh A (2011). The impact of eHealth on the quality and safety of health care: a systematic overview. PLoS Med.

[16] Ward R (2013). The application of technology acceptance and diffusion of innovation models in healthcare informatics. Health Policy and Technology.

[17] Harrison MI, Koppel R, Bar-Lev S (2007). Unintended consequences of information technologies in health care-an interactive sociotechnical analysis. J Am Med Inform Assoc.

[18] Kaplan B, Harris-Salamone KD (2009). Health IT success and failure: recommendations from literature and an AMIA workshop. J Am Med Inform Assoc.

[19] Kutney-Lee A, Sloane DM, Bowles KH, Burns LR, Aiken LH (2019). Electronic Health Record Adoption and Nurse Reports of Usability and Quality of Care: The Role of Work Environment. Appl Clin Inform.

[20] Harris PA, Taylor R, Thielke R, Payne J, Gonzalez N, Conde JG (2009). Research electronic data capture (REDCap)-a metadata-driven methodology and workflow process for providing translational research informatics support. J Biomed Inform.

[21] Clarke V, Braun V (2013). Successful qualitative research: A practical guide for beginners.

[22] Carlson RV, Boyd KM, Webb DJ (2004). The revision of the Declaration of Helsinki: past, present and future. Br J Clin Pharmacol.

[23] Crow A A helping hand for clinicians: how clinical help desks can improve care, increase adoption, and contain costs 2018. Becker's Health IT.

[24] Gandhi TK, Graydon-Baker E, Huber CN, Whittemore AD, Gustafson M (2005). Closing the Loop: Follow-up and Feedback in a Patient Safety Program. The Joint Commission Journal on Quality and Patient Safety.

[25] HIMSS Usability Task Force (2011). Promoting Usability in Health Organizations: initial steps and progress toward a healthcare usability maturity model. Health Information and Management Systems Society.

[26] Olsen LA, Aisner D, McGinnis JM, Institute of Medicine (US) Roundtable on Evidence-Based Medicine (2007). The Learning Healthcare System: Workshop Summary.

