# Supplementary figures and images for: Beyond Getting Rid of Stupid Stuff in the Electronic Health Record (Beyond-GROSS): Protocol for a User-Centered, Mixed-Method Intervention to Improve the Electronic Health Record System
Source: JMIR Res Protoc. 2021 Mar 16;10(3):e25148. doi: 10.2196/25148 (PMC8294464; doi:10.2196/25148)

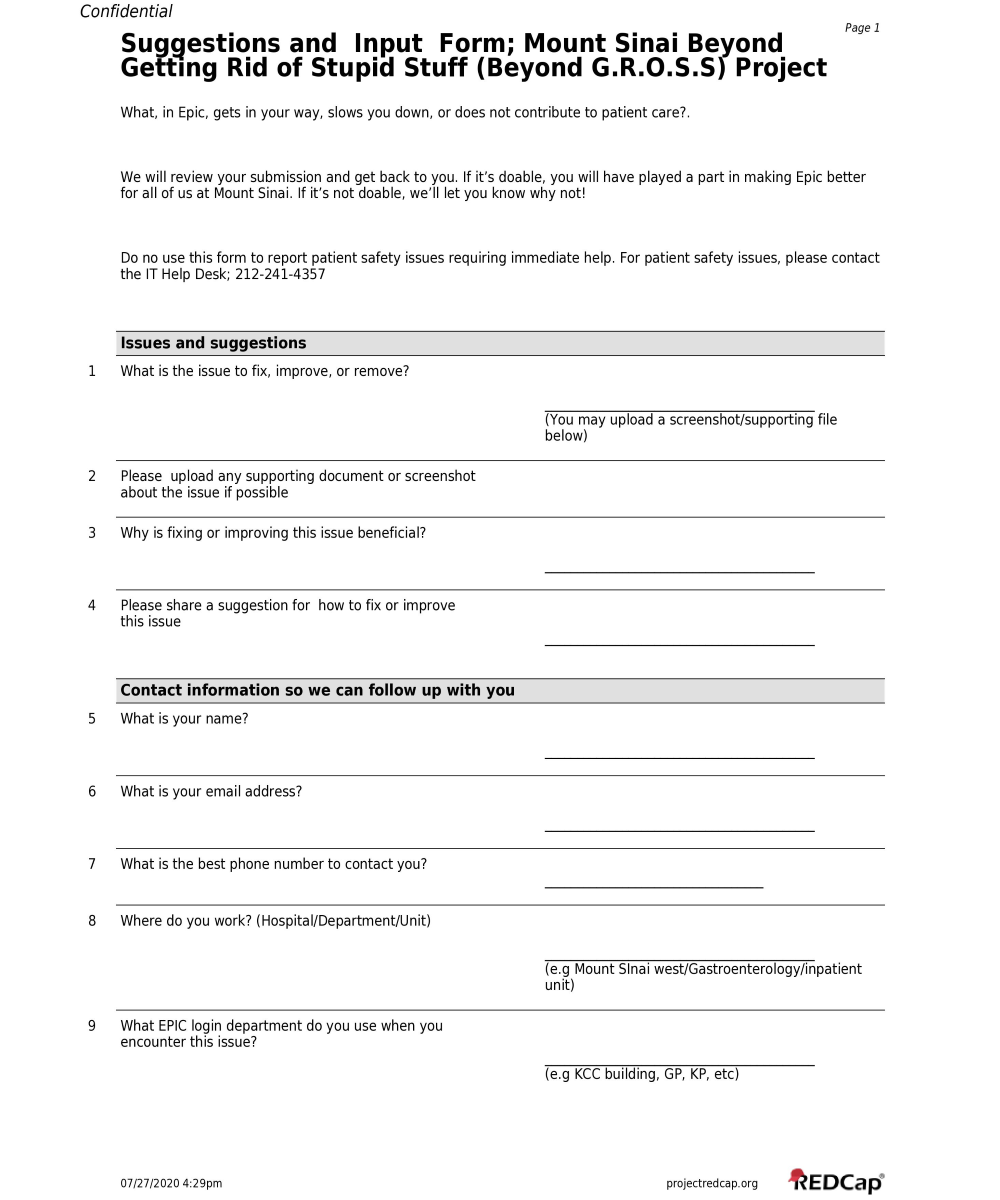

Supplement: Multimedia Appendix 1 [file resprot_v10i3e25148_app1.png]

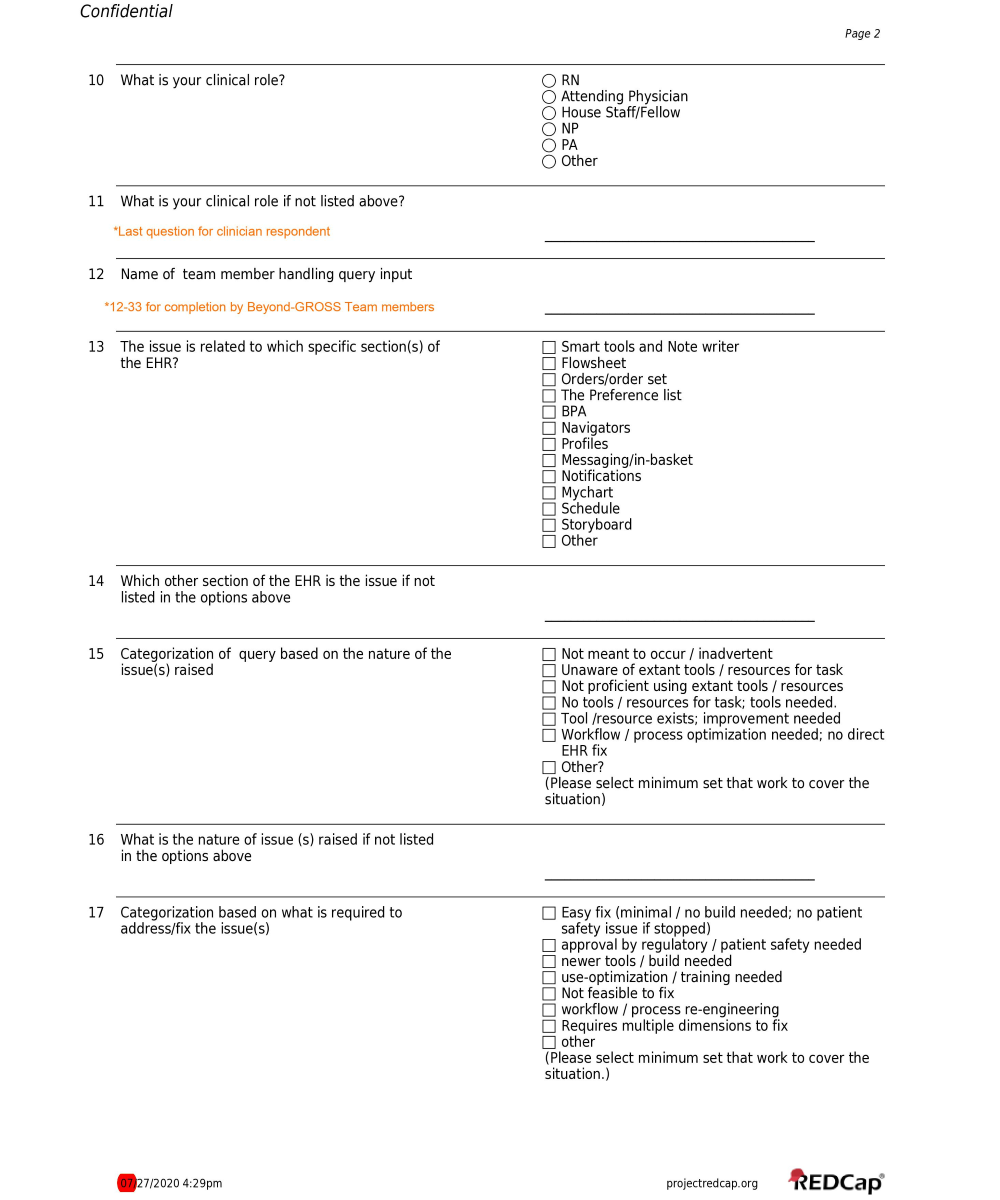

Supplement: Multimedia Appendix 2 [file resprot_v10i3e25148_app2.png]

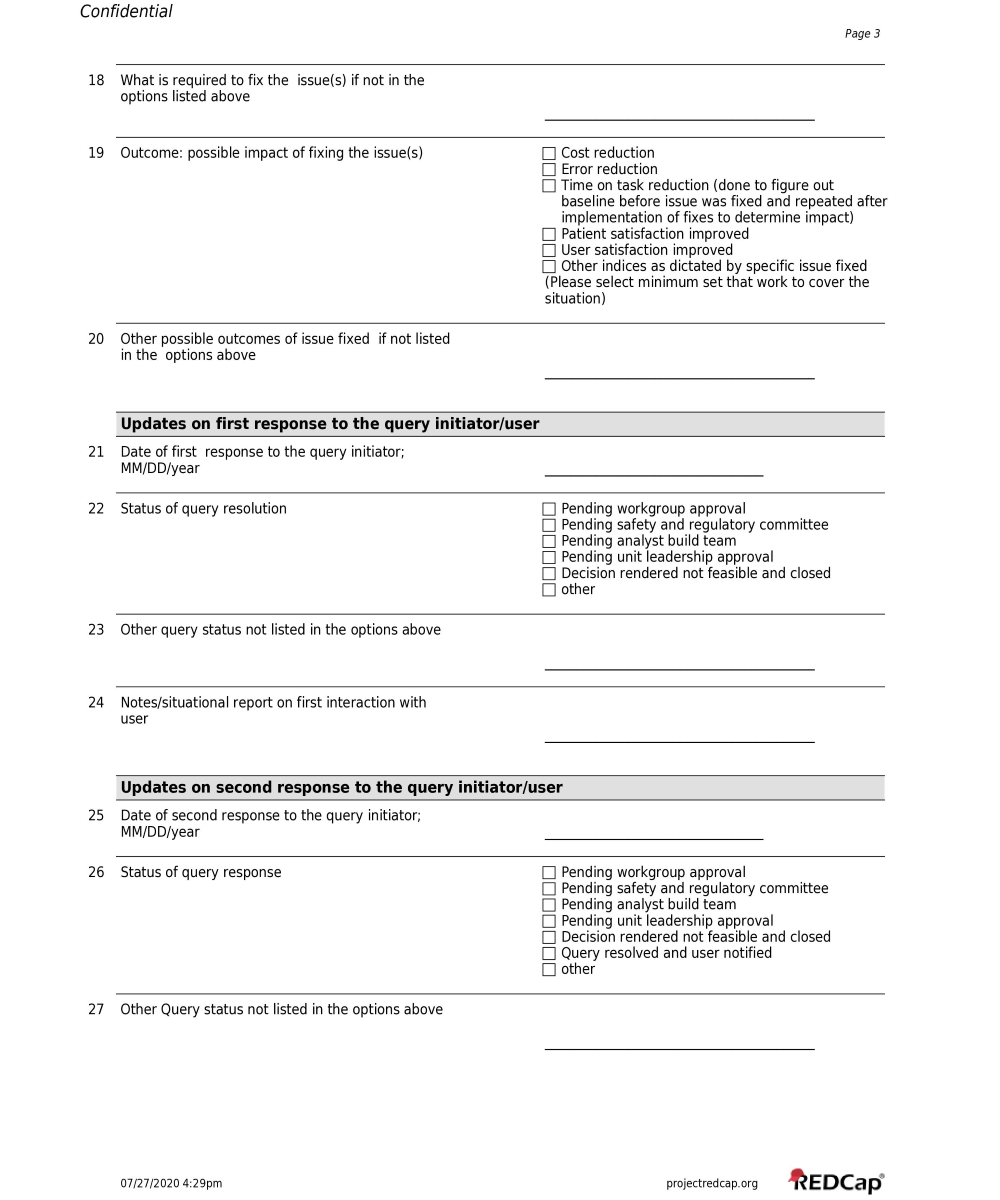

Supplement: Multimedia Appendix 3 [file resprot_v10i3e25148_app3.png]

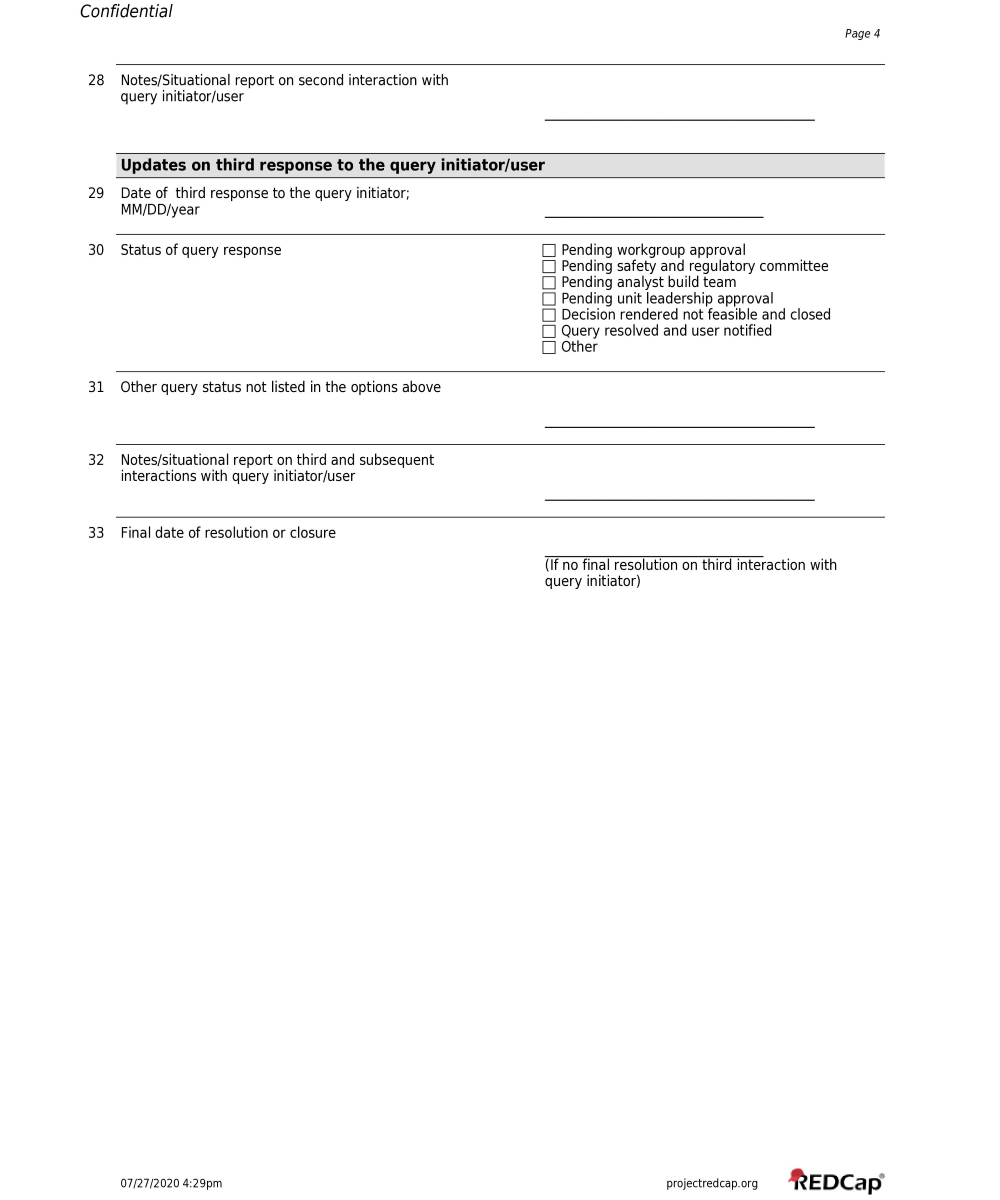

Supplement: Multimedia Appendix 4 [file resprot_v10i3e25148_app4.png]
